# Laparoscopic-Assisted Resection for an Extra-gastrointestinal Stromal Tumor Arising From the Mesentery of the Small Intestine: A Case Report

**DOI:** 10.7759/cureus.60897

**Published:** 2024-05-23

**Authors:** Ayano Takahashi, Kentaro Inada, Yusuke Kitani, Takayoshi Koseki, Tsuyoshi Maeshiro

**Affiliations:** 1 Department of Gastrointestinal Surgery, Saitama Prefectural Cancer Center, Saitama, JPN; 2 Department of Surgery, Tokyo Metropolitan Bokutoh Hospital, Tokyo, JPN

**Keywords:** extra-gastrointestinal stromal tumor, uterine myoma, c-kit, laparoscopy, gastrointestinal stromal tumor

## Abstract

Gastrointestinal stromal tumors (GISTs) are mesenchymal tumors that arise in the muscular or submucosal layers of the gastrointestinal tract. Extra-gastrointestinal stromal tumors (EGISTs) are rare primary entities that develop outside the digestive tract which are histologically and immunologically similar to GISTs.

We present the case of a 52-year-old female diagnosed with a primary EGIST arising in the small bowel mesentery four months after undergoing hormone therapy for multiple uterine myomas. Transvaginal ultrasonography and MRI revealed a pelvic mass suspected to be a GIST. The patient was treated with laparoscopic-assisted partial resection of the small bowel. Histopathological examination of the surgical specimen confirmed the diagnosis of an EGIST. Imatinib treatment was initiated, and no clinical evidence of recurrence or metastasis was detected postoperatively.

Because EGISTs are extremely rare, the differences between EGISTs and GISTs, the degree of malignancy, and prognosis have not been fully investigated. Further studies are needed to accumulate additional cases. The present case shows that laparoscopic-assisted excision can be successfully used to manage EGISTs.

## Introduction

Gastrointestinal stromal tumors (GISTs) are mesenchymal tumors that arise in the muscular or submucosal layers of the gastrointestinal tract. Several reports have been published about extra-gastrointestinal stromal tumors (EGISTs). EGISTs, which are histologically and immunologically similar to GISTs, are rare primary entities that develop outside the digestive tract [1-3]. We report a case of laparoscopic surgery for a pelvic mass that was suspected to be a uterine myoma but was finally diagnosed as an EGIST postoperatively.

## Case presentation

The patient was a 52-year-old female who had been undergoing hormone therapy for multiple uterine myomas at a gynecological center for four months. Transvaginal ultrasonography and magnetic resonance imaging (MRI) revealed a pelvic mass, which was suspected to be a GIST. The patient had no subjective symptoms. Other than treatment for multiple uterine myomas, the patient had a past medical history of laparoscopic left nephrectomy for a ruptured left renal cyst two and a half years prior and had been diagnosed with left ureteral junction stenosis more than 20 years prior. She was taking relugolix (a gonadotropin-releasing hormone antagonist) and herbal medicine orally. She had no history of alcohol or tobacco use and no relevant family history.

**Figure 1:**
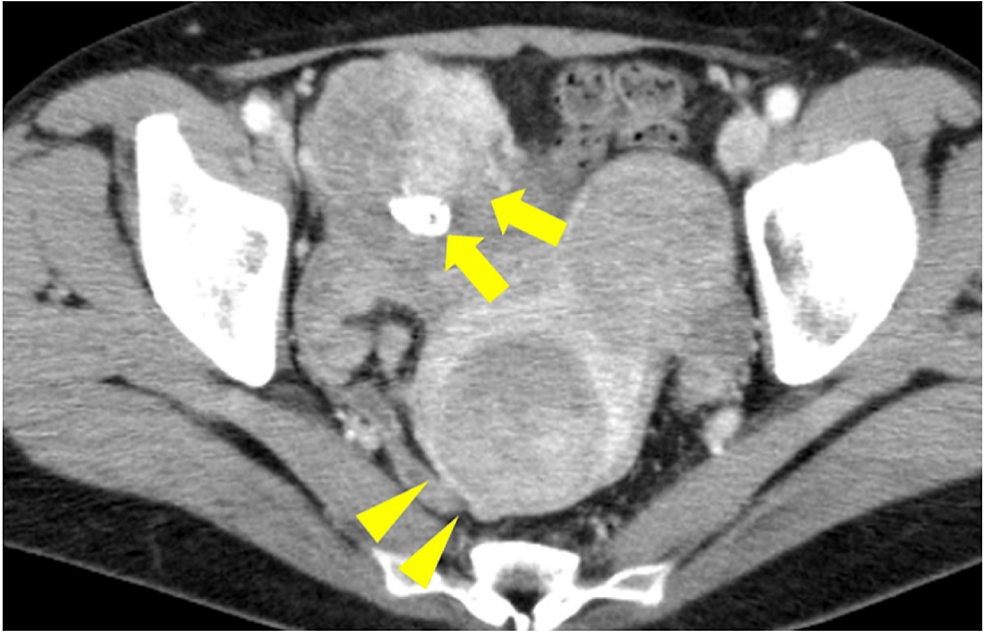
The preoperative abdominopelvic contrast CT image (axial view). A 50 x 68 mm mass lesion (arrow) with a heterogeneous contrast effect can be observed on the right side of the pelvis. The tumor had moved ventrally and grown in size compared to prior imaging (Figure 3). Arrowheads represent uterine myomas that have already been noted.

**Figure 2:**
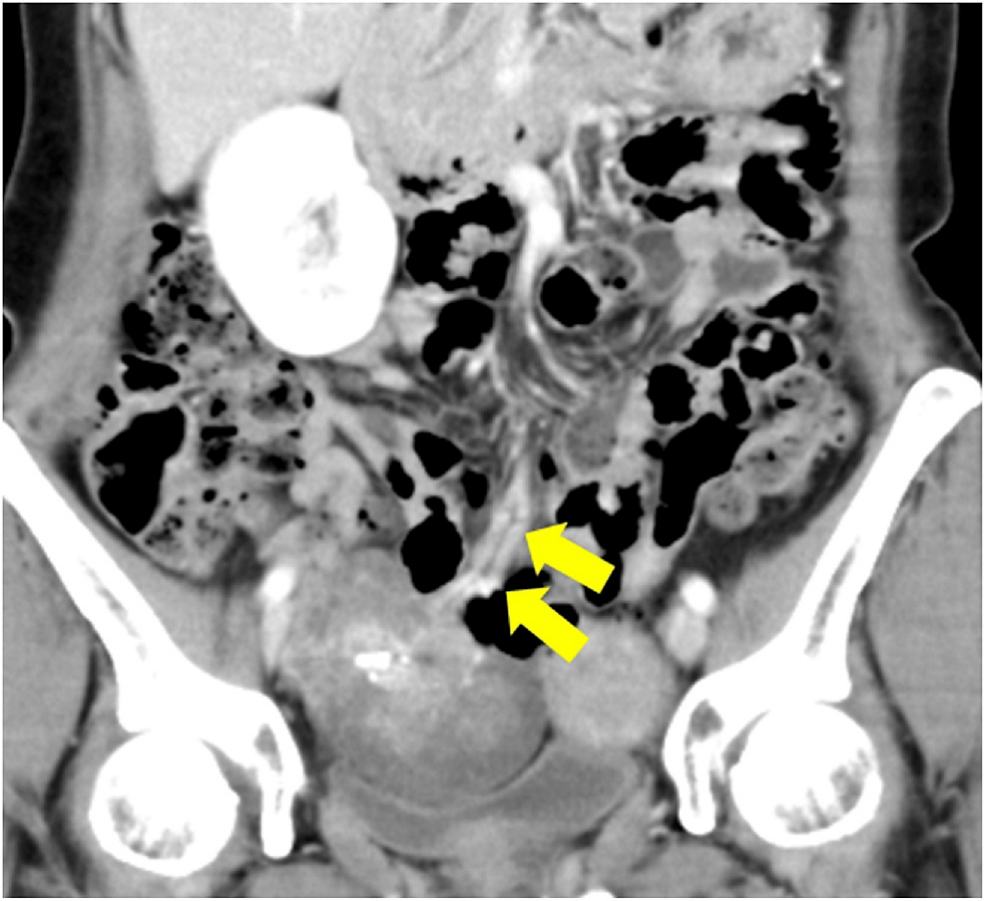
Preoperative abdominopelvic contrast CT image (coronal view). A peripheral branch of the SMV mesenteric vein flowed into the mass. SMV: Superior mesenteric vein

**Figure 3:**
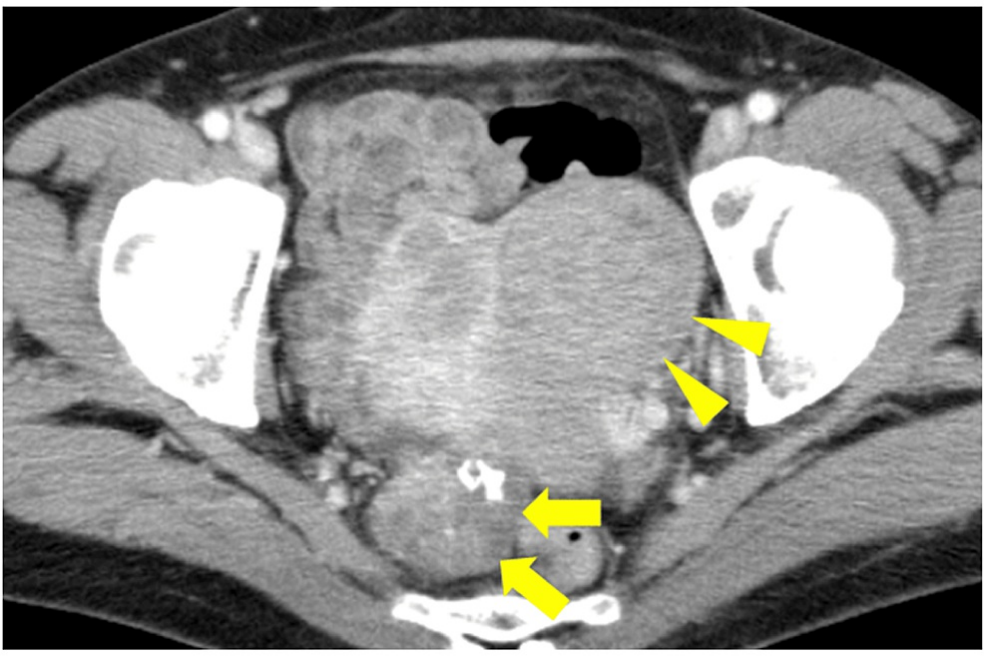
Abdominal pelvic contrast CT tomography performed two years prior to urological surgery. A mass with calcification (arrow) can be observed in the right dorsal pelvis. A uterine myoma observed on the ventral side (arrowhead) is thought to be the same as the uterine myoma at presentation (Figure 1).

**Figure 4:**
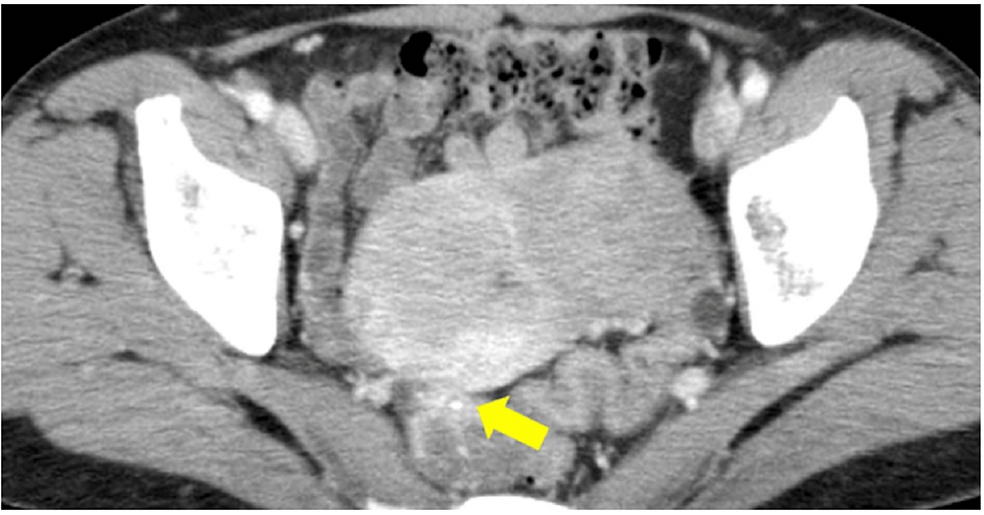
Abdominal pelvic contrast CT tomography performed two years prior to urological surgery. Retrospectively, the peripheral branches of the SMV were shown to flow into the mass after the point indicated by the arrow. SMV: Superior mesenteric vein

**Figure 5:**
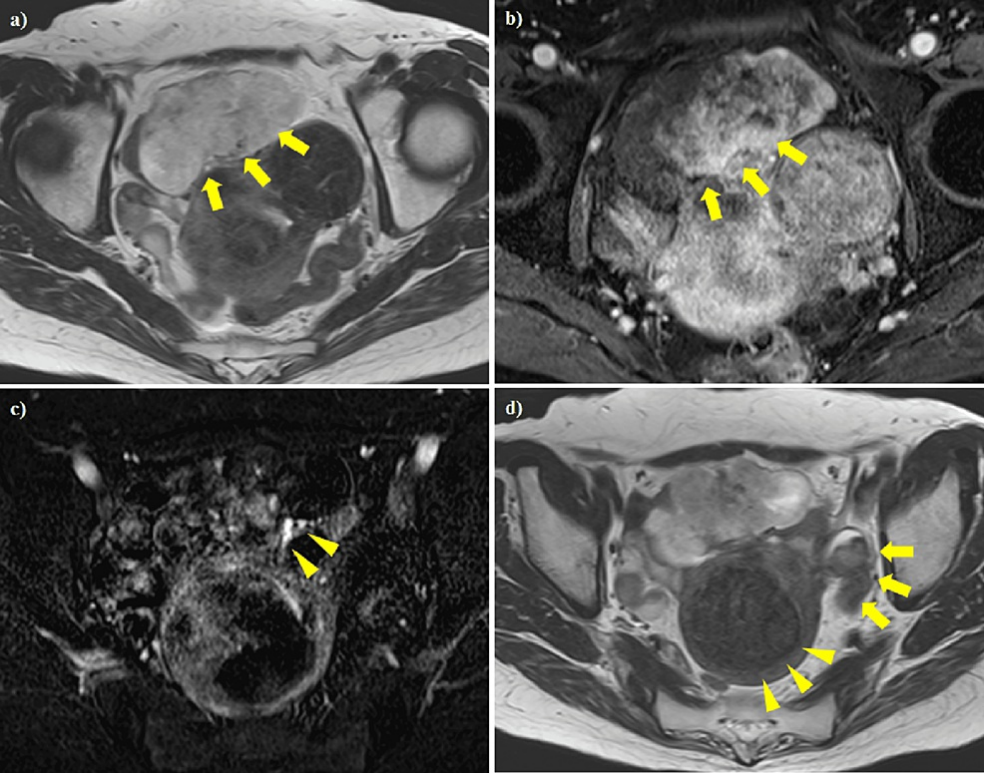
Abdominal pelvic contrast-enhanced MRI. (a) T2-weighted images revealing a heterogeneous lobulated mass. (b) Contrast enhancement. The interior of the tumor showed a progressive contrast effect. There was poor contrast, consistent with a low-signal area in the T2-weighted images. (c) Contrast enhancement. The peripheral branches of the SMV entered the mass. (d) T2-weighted images. The left ovary (arrow) was visible, but the right ovary could not be identified. A uterine myoma (arrowhead) was observed. SMV: Superior mesenteric vein

On clinical examination, her vital parameters were within normal limits; however, abdominal examination revealed a well-defined lower abdominal mass with no pain on palpation. Surgical scars were observed on the left side of the abdomen during urological surgery. Laboratory test findings, including full blood count, electrolytes, liver function tests, and tumor markers (carcinoembryonic antigen and carbohydrate antigen 19-9), were within normal limits. Chest and abdominal radiographs were unremarkable. Contrast-enhanced computed tomography (CT) revealed a large abdominal and well-defined mass (50×68 mm) with a heterogeneous contrast effect on the right side of the pelvis (Figures 1, 2). Compared to CT performed three years earlier, before urologic surgery (Figures 3, 4), the location of the lesion had shifted, and its size tended to increase. The tumor was suspected to be located on the mesentery due to its proximity to one of its edges with the peripheral portion of the superior mesenteric vein (SMV). The right ovary could not be identified, and there were no significant changes in the previously noted uterine myomas. Contrast-enhanced MRI (Figures 5a-5d) showed a mass with heterogeneous internal contrast on the ventral side of the uterine body. Similar to that in the CT scan, the right ovary was difficult to identify. Based on these imaging findings, we considered a primary mesenteric tumor (EGIST of the small mesentery or desmoid) or a right ovarian tumor as differential diagnoses, with the former considered more likely based on imaging findings of the peripheral SMV. The CT scan at the time of urological surgery showed a pelvic mass with calcification, which was thought to be a uterine myoma; however, retrospectively, the lesion was present from this point, and its size had increased between the two imaging techniques. As the origin of the tumor was not clear, we first observed the abdominal cavity by laparoscopy before deciding on a surgical plan. Pretreatment, it was decided that the tumor originated from the small intestinal mesentery, partial resection of the small intestine would be performed; if it originated from the ovary, surgery would be performed by the gynecology department.

**Figure 6:**
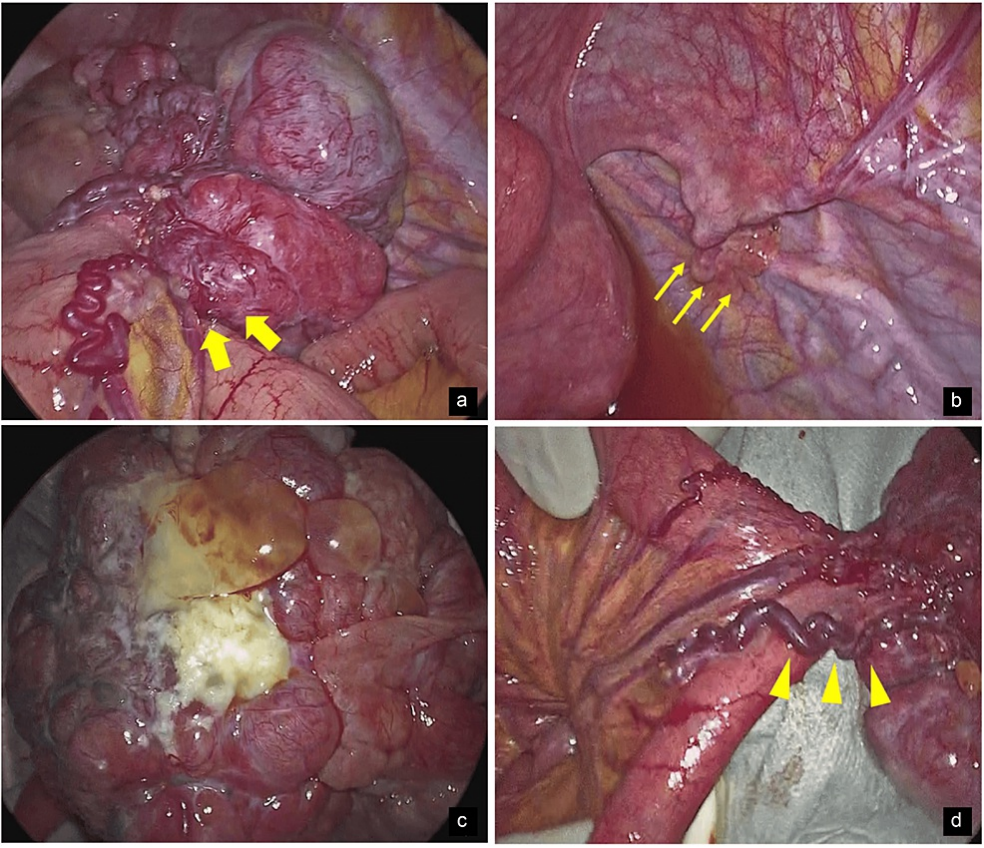
Intraoperative findings. (a and b) The tumor fell into the pelvic cavity. Since the tumor was contiguous with the wall of the small intestine (arrow) and a normal right ovary could be seen (thin arrow), we considered that it was not an ovarian tumor, but instead, a tumor originating from the small intestine or mesentery. (c and d) Observation after removal of the tumor from the body. The tumor retracted a portion of the contralateral mesenteric wall of the small intestine, and inflamed mesenteric vessels (arrowheads) flowed into the tumor. The tumor surface was uneven and irregular with prominent hypervascularization, some of which was white. Mucus leakage was also observed.

**Figure 7:**
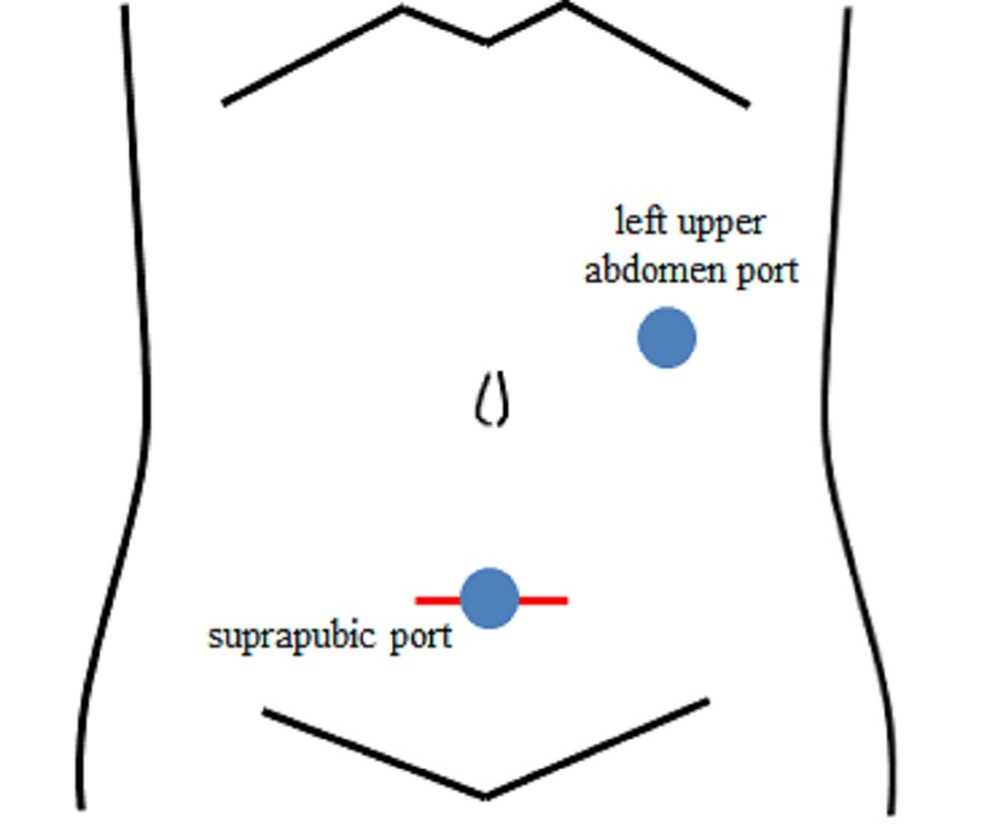
Schema of port locations. A 5 mm port was inserted above the pubis and another 5 mm port was inserted into the left upper abdomen (adjacent to the urologic surgery scar) for a total of two ports. Finally, the suprapubic port wound was widened with a Pfannenstiel incision. The tumor was removed from the body for observation.

**Figure 8:**
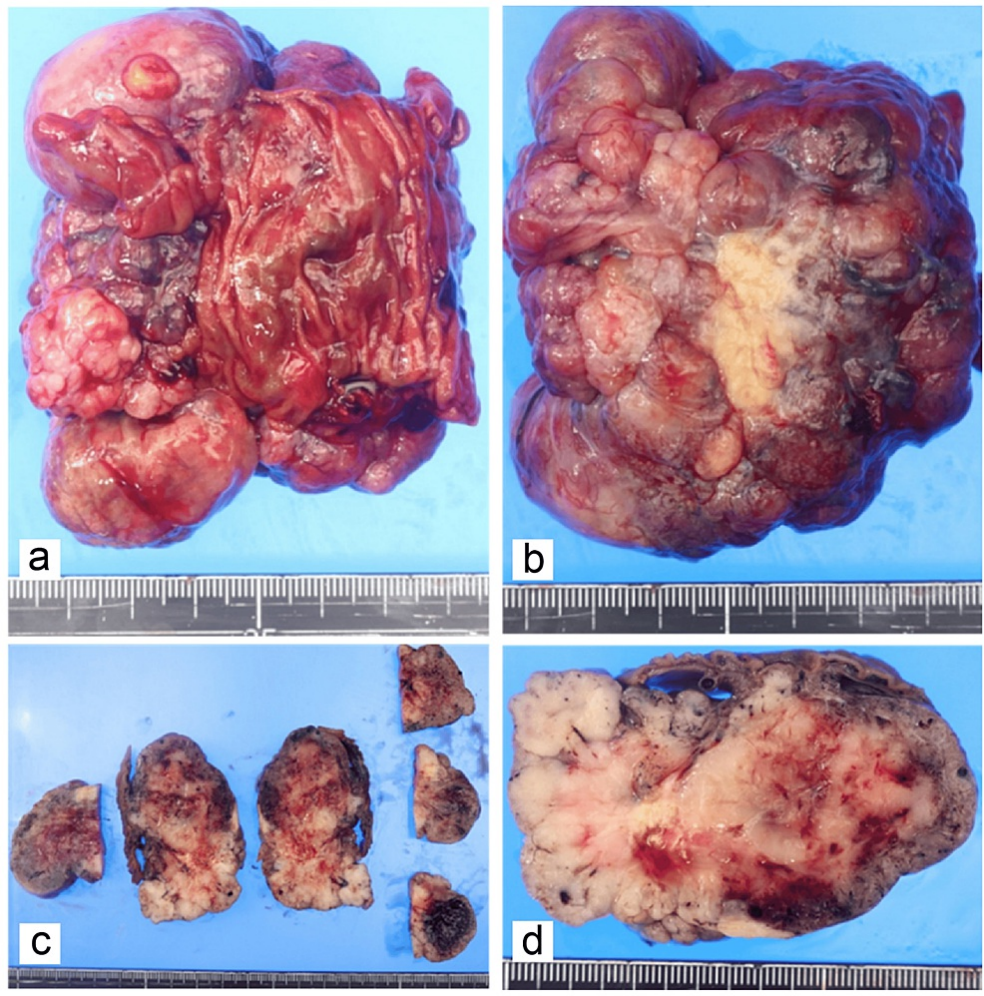
Gross findings of the resected specimen. (a-d) The resected small intestine was 4.7 cm long. A mass 8.5 x 8.0 x 4.0 cm in size was observed on the serosal side of the small intestine, but there was no exposure to the mucosal surface of the small intestine.

The surgical findings (Figure 6) were as follows: We started the operation laparoscopically. A 5 mm port was inserted above the pubis and another 5 mm port was inserted into the left upper abdomen (adjacent to the urologic surgery scar) for a total of two ports (Figure 7). Yellow transparent ascites was observed in the Douglas fossa. This information was collected and submitted for cytological diagnosis. The mass was mobile and contiguous with the wall of the small intestine, and a normal right ovary was identified; therefore, we considered it to be a tumor of the small intestine or small mesentery. The tumor was moved to just below the suprapubic port. After widening the port wound with a Pfannenstiel incision, the tumor was removed from the body for observation. The tumor had retracted part of the small intestinal wall on the opposite mesenteric side, and the enlarged mesenteric vessels flowed into the tumor. The tumor surface was irregular with prominent hypervascularization, some of which had turned white, and mucous leakage was observed. We performed laparoscopic-assisted partial resection of the small intestine. The resected small intestine was 4.7 cm long, and an 8.5 x 8.0 x 4.0 cm mass was observed on the serosal side of the small intestine. There was no tumor exposure to the mucosal surface of the small intestine, and the divided surface was mildly lobulated, milky to grayish-white, with mixed hemorrhage, and some calcification and ossification (Figure 8). Douglas fossa ascites cytology revealed no malignancy.

**Figure 9:**
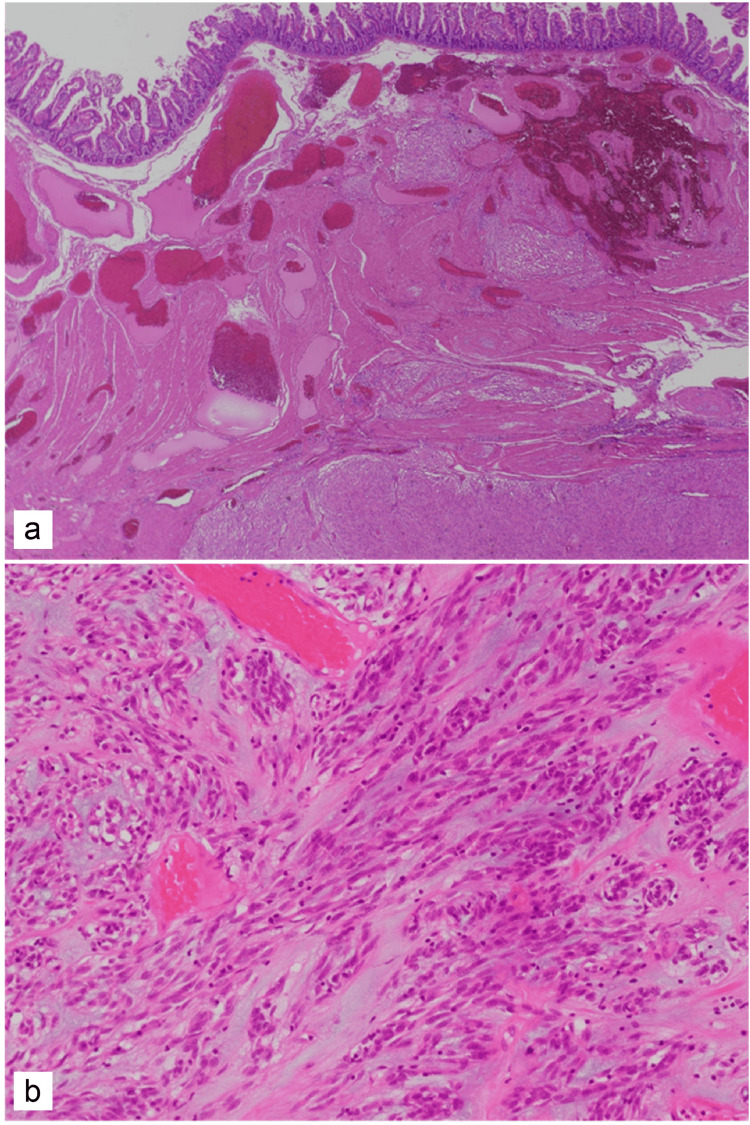
Histopathological findings. (a and b) Hematoxylin-Eosin (H-E) staining. The spindle-shaped tumor cells were enlarged and had invaded the subserosa and muscularis propria.

**Figure 10:**
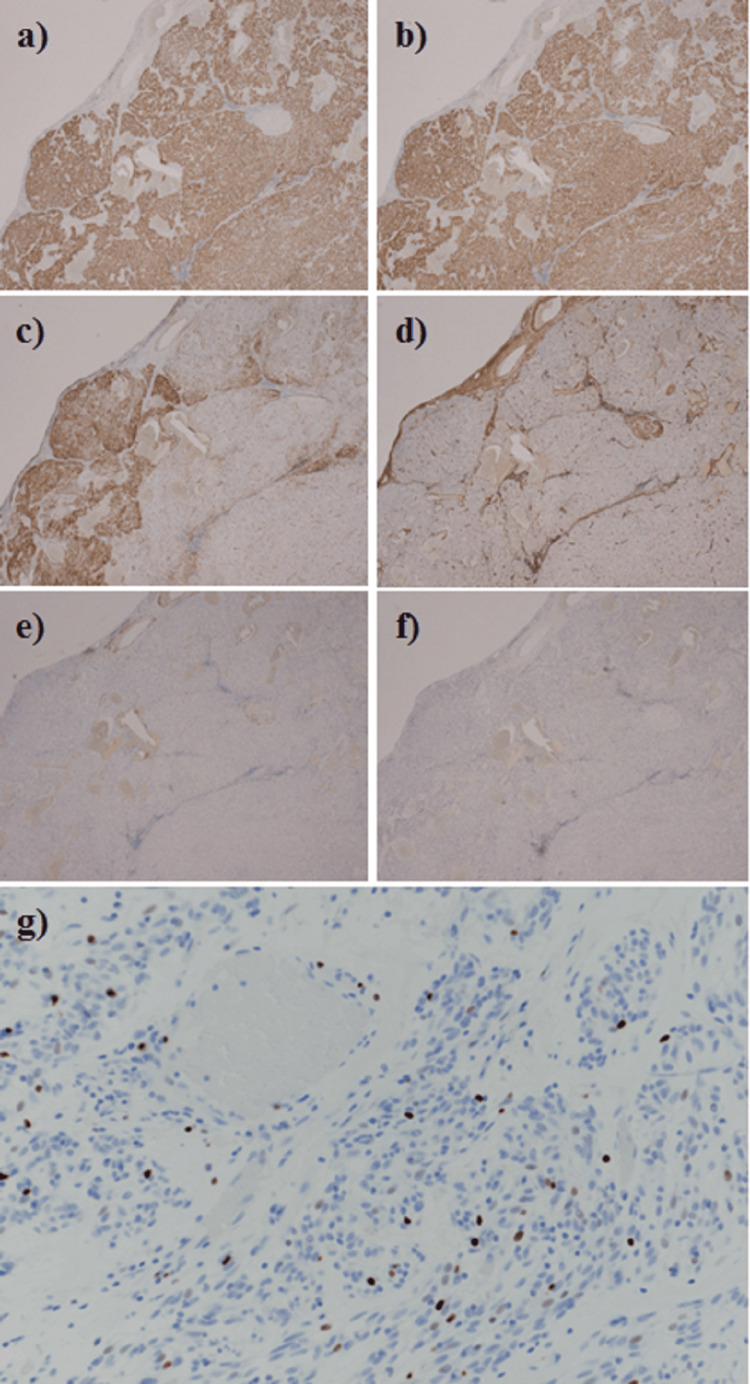
Immunohistochemistry. (a) Discovered on GIST Protein 1 (DOG-1) staining was positive. (b) CD117 (c-kit) was positive. (c) CD34 was partially positive. (d) The patient tested negative for desmin. (e) S-100 was negative. (f) α-SMA was negative. (g) The Ki-67 antigen positivity rate was approximately 8%.

Histopathological findings (Figures 9, 10) were as follows: Tumor cells grew mainly in the submucosa, with some invading the muscularis propria. The tumor cells were spindle and round with enlarged nuclei, bundled, and follicular with a background of myxomatous stroma, vitreous fibrosis, and hemorrhage, some of which proliferated in a lobulated pattern. Lymphocytic infiltration within the lesion, numerous intervening blood vessels, and incomplete bone formation was observed. Immunohistochemistry revealed positive results for Discovered on GIST Protein 1 (DOG-1), CD117 (c-kit), and CD34, and negative results for Desmin, S-100 protein (S-100), and α-Smooth muscle actin (α-SMA), which were similar to the immunological features of GISTs. The proliferation of tumor cells mainly on the serosal side of the small intestine and the fact that blood vessels in the mesentery crossed the normal small intestine and flowed into the tumor led to the diagnosis of small intestinal invasion of a primary EGIST in the mesentery. The Ki-67 antigen positivity rate was approximately 8%. No evident vascular invasion was observed. Resection margins were negative. In the modified Fletcher classification, the tumor was classified as high-risk based on the tumor diameter of 8 cm, mitotic image 6-10/50 high-power field (HPF), and primary site, except for the stomach. According to the TNM classification, the diagnosis was pT3NXM0, pStage IIIB.

Postoperatively, the patient started eating on the third postoperative day and was discharged on the seventh postoperative day. Considering the risk of recurrence, the patient was treated with postoperative adjuvant therapy with imatinib and followed-up every six months.

## Discussion

A GIST is a stromal cell tumor first reported in 1983 by Mazur et al. [4]. Hirota et al. reported KIT protein expression and gain-of-function mutations in the c-kit gene in GIST, which were defined as tumors expressing KIT and composed of spindle-shaped or epithelioid tumor cells [5]. GISTs have been shown to arise from interstitial cells of Cajal (ICCs) or undifferentiated mesenchymal cells that differentiate into ICCs in the gastrointestinal muscularis propria. Because ICCs function as pacemakers for gastrointestinal peristalsis and are located in the muscularis propria of the gastrointestinal tract, especially around the ganglion cells of the intermuscular plexus, GISTs most commonly occur in the muscularis propria of the gastrointestinal tract [6]. However, tumors with histological and immunological features similar to GISTs have been reported to arise outside the gastrointestinal tract and are called EGISTs. Cases of EGISTs in the omentum, mesentery, and retroperitoneum have also been reported. EGISTs are extremely rare, accounting for approximately 5% of all GISTs [7]. EGISTs occur when ICCs do not originally exist, and their origin is still unknown; however, it is speculated that they may be derived from ICC-like cells outside the gastrointestinal tract. Sakurai et al. previously reported the presence of CD117-positive ICC-like cells just below mesothelial cells in the omentum, which could be the cell of origin of EGISTs [8]. In an immune-histological study of EGISTs arising in the mesentery and omentum [2], 13 of 14 cases were CD117 positive and 15 of 23 cases were α-SMA positive. Based on these results, the authors concluded that progenitor cells differentiating into ICCs and smooth muscle cells may be the developmental origin of EGISTs.

Since mutations in the c-kit and platelet-derived growth factor receptor alpha (PDGFRA) genes have been associated with the development of GISTs, corresponding immunohistochemistry has been shown to be useful for diagnosis [5]. CD117 (c-kit) and CD34 antibodies were detected in more than 95% and 70% of cases, respectively. Other, less frequent classifications have also been identified; for example, α-SMA is positive in 30%, S-100 in 5%, Desmin in 2%, and cytokeratin in 2% of cases [9]. EGISTs showed the same immunohistological features as GISTs.

Based on a previous study, Yamamoto et al. advocated the following three diagnostic criteria for EGISTs [10]: (1) tumor arising from soft tissues in the abdominal cavity, retroperitoneum, or pelvis, and not continuous with the gastrointestinal tract; (2) showing histological features similar to GISTs; and (3) kit-positivity on immunohistochemistry. In our case, items 2 and 3 were satisfied. Regarding item 1, tumor cell infiltration in the subserosa and muscularis propria of the small intestine made differentiation from an extra-mural type GIST difficult. However, based on the fact that tumor cell growth was mainly on the serosal side of the small intestine and that blood vessels in the mesentery overcame the normal small intestine and flowed into the tumor (Figure 6d), we considered the tumor to be an EGIST, which originated in the small intestinal mesentery, invaded the small intestine, and developed continuity with the digestive tract as the tumor grew.

EGISTs are less symptomatic than GISTs in the gastrointestinal tract, are difficult to diagnose endoscopically, and are often detected after the tumor has grown in size [11,12]. Cases in which the tumor was discovered only after internal necrosis, hemorrhage, or spontaneous rupture due to tumor enlargement have been reported. However, the prognosis and grade of malignancy have not been fully evaluated, and the treatment is generally the same as that for GISTs. Surgery is the first choice of treatment in the absence of metastasis. Due to the large tumor size in most cases, risk classification using the Modified Fletcher Classification places patients in the high-risk group regardless of the fission pattern, and postoperative adjuvant therapy is therefore required. In our case, the tumor diameter was 8 cm, the fission pattern was 6-10/50 HPF, and the primary site was not the stomach, placing the patient in the high-risk group according to the modified Fletcher classification. Therefore, the patient was started on postoperative adjuvant therapy with imatinib for three years after discharge from the hospital.

Although many cases of laparoscopic resection have been reported for surgical treatment, there is no consensus on the size of tumors suitable for laparoscopic surgery. The Japanese guidelines for the treatment of GISTs [13] state that laparoscopic surgery is weakly recommended when the tumor diameter is 5 cm or larger. There have been three previous reports comparing the outcomes of laparotomy and laparoscopy for GISTs larger than 5 cm in diameter [14-16]. In all three studies, short-term outcomes, including blood loss, operative time, perioperative complications, and hospital stay, were comparable or better for laparoscopic surgery. In addition, long-term outcomes such as recurrence-free survival and overall survival were comparable or better for laparoscopic surgery. Furthermore, there are currently no data suggesting a difference in the incidence of positive resection margins or tumor rupture. However, it should be noted that most of the cases reviewed in these studies had tumor diameters of 8 cm or less, and laparotomy is often selected in actual clinical situations when the diameter exceeds 8 cm.

In our case, the diameter of the tumor was > 8 cm; however, considering the location of the tumor based on preoperative imaging, the camera port was placed on the pubic bone, and the tumor was removed using the wound. This allowed us to perform a minimally invasive surgery without capsular damage. Although laparoscopic surgery is not beneficial when the tumor size is too large to require a skin incision length as long as that of laparotomy, or when the working space in the abdominal cavity cannot be secured, this report suggests that laparoscopic surgery can be a useful technique for this type of device.

## Conclusions

Herein, we reported a case of laparoscopic surgery for an EGIST arising in the mesentery of the small intestine. For GISTs, including EGISTs, it is sometimes difficult to determine the organ of origin preoperatively, and differentiation from other diseases, such as ovarian tumors or uterine myomas, may be required, as in this case. In such cases, the ability to observe the abdominal cavity and obtain information on the site of origin and the presence or absence of invasion into other sites, as well as to determine the diagnosis and surgical procedure, are considered advantages of laparoscopic surgery. Because EGISTs are extremely rare, the differences between EGISTs and GISTs, as well as the degree of malignancy and prognosis, have not been fully evaluated. Therefore, treatment is currently provided in accordance with the guidelines for the treatment of GISTs. Further studies are required based on the accumulation of additional cases; however, the present case suggests that laparoscopic resection followed by chemotherapy can achieve good results.
